# Effectiveness and Cultural Adaptation of Parenting Interventions for South Asian Families: A Mixed-Methods Systematic Review Using Bernal’s Ecological Validity Model

**DOI:** 10.3390/children13010086

**Published:** 2026-01-06

**Authors:** Aleena Syed, Usman Arshad, Karina Lovell, Nusrat Husain, Alexander Hodkinson, Maria Panagioti

**Affiliations:** 1 Division of Psychology and Mental Health, University of Manchester, Manchester M13 9PG, UK; 2 Division of Nursing and Midwifery, University of Manchester, Manchester M13 9PG, UK; 3 Division of Population Health, Health Services, Research and Primary Care, University of Manchester, Manchester M13 9PG, UKmaria.panagioti@manchester.ac.uk (M.P.)

**Keywords:** parenting, child development, culturally adapted parenting programs, South Asians

## Abstract

**Highlights:**

**What are the main findings?**

- Culturally adapted parenting interventions improved child outcomes, parenting knowledge, and psychological wellbeing among South Asian families.
- Deeper cultural adaptation was linked to stronger and more consistent effects on children’s cognitive development.

**What are the implications of the main findings?**

- Policymakers and practitioners should prioritise the development and evaluation of culturally grounded parenting interventions that integrate psychosocial support for low mood, tailored to South Asian contexts.
- Future research should employ robust study designs and clearly document adaptation processes to strengthen the evidence base.

**Abstract:**

Background: Although parenting interventions are effective in improving parenting practices and child development, most are developed within Western cultural frameworks that may not align with South Asian collectivist values and family structures. The extent to which cultural adaptation influences the effectiveness of parenting interventions in South Asian populations remains unclear. Aim: To systematically review the effectiveness of parenting interventions on child developmental outcomes, parenting outcomes, and parental health among South Asian families, and to examine whether the depth of cultural adaptation, assessed using Bernal’s Ecological Validity Model (EVM), is associated with intervention effectiveness. Methods: A systematic review and meta-analysis were conducted. We systematically searched CINAHL, MEDLINE, Science Direct, PsychINFO, PubMed, and Cochrane library. Data were extracted from six electronic databases up to August 2023. Quality and risk of bias were appraised using the Revised Cochrane Risk of Bias Tool for Randomized Trials for the quantitative studies and the Critical Appraisal Skill Program (CASP) checklist for the qualitative studies. Results: Seventeen studies (fifteen quantitative, two qualitative) involving 8088 participants were included; ten studies contributed data to meta-analysis. Parenting interventions were associated with moderate improvements in parenting knowledge (SMD = 0.51, 95% CI 0.25 to 0.76) and small improvements in parental involvement (SMD = 0.36, 95% CI 0.00 to 0.72). Significant reductions in parental depression (SMD = −0.77, 95% CI −1.20 to −0.34) and disability symptoms (SMD = 0.82, 95% CI 0.68 to 0.96) were observed, though effects on post-natal depression (SMD = 0.15, 95% CI −1.00 to 1.30) and physical quality of life (SMD = −0.27, 95% CI −1.22 to 1.75) were non-significant. For children, large improvements were found in cognitive (SMD = 0.84–1.48), language (SMD = 0.79, 95% CI 0.25 to 1.33), and social development (SMD = 0.54, 95% CI 0.16 to 0.91), but not in emotional or motor development. Sensitivity analyses indicated larger effects for studies demonstrating deeper cultural adaptation. Qualitative findings highlighted maternal empowerment, improved mental wellbeing, and the importance of family support and culturally congruent facilitators for engagement. Overall certainty of evidence was rated as low due to high heterogeneity, risk of bias, and imprecision. Discussion: Culturally adapted parenting interventions show promising benefits for parenting practices, parental mental health, and child developmental outcomes among South Asian families, particularly when adaptations extend beyond surface-level changes. However, evidence quality is low and inconsistent, highlighting the need for more rigorous trials and clearer reporting of cultural adaptation to optimize effectiveness.

## 1. Introduction

Developmental delays in children under the age of five are defined as delays in one or more developmental domains, including gross motor, fine motor, speech and language, and social development [1]. According to the Lancet series, approximately 53% of children under five in South Asia are at risk of not reaching their full developmental potential [2]. Poor maternal health and high stress levels often shaped by socio-cultural and economic challenges are consistently linked to suboptimal child development outcomes in low- and middle-income countries [3].

Gentle, responsive parenting and early cognitive stimulation are foundational to healthy child development. In contrast, their absence manifested as emotional unavailability, harsh discipline, or neglect has been strongly associated with developmental delays in early childhood [4]. Therefore, early parenting interventions that promote positive parent–child interactions are essential [5].

Parenting programs are designed around Western parenting norms, including encouraging independence and personal agency [6], which may not align with the collectivist values and hierarchical family practices prevalent across South Asian countries. While these countries have cultural and social differences, such as variations in religious practices, gender norms, and parenting roles, they share strong commonalities in extended family involvement and traditional caregiving practices [7]. Moreover, traditional caregiving emphasized extended family involvement and collective child-rearing, 21st-century parenting in South Asia increasingly incorporates responsive practices and early cognitive stimulation, influenced by urbanization, education, and exposure to global norms. Compared to the Global North, where individualism and independence are often prioritized, South Asian parenting continues to balance these emerging practices with longstanding cultural values [8].

Bernal [9] defines cultural adaptation as the process of modifying evidence-based interventions to align with the values, beliefs, language, and contextual realities of a target population while maintaining core components that ensure effectiveness. Established parenting interventions have been widely recognized for enhancing emotional availability, child stimulation, and strengthening the parent–child relationship. Numerous studies have shown that such interventions enhance parental knowledge, promote positive parenting styles, and support skill development [10]. Consequently, they have been associated with improvements in children’s cognitive, motor, language, and socio-emotional development, and overall well-being [11].

However, South Asian culture is known for its unique parenting practices such as authoritarian parenting, emotional restraint, and strong joint family influences on raising the children [12]. Previous reviews have primarily examined parenting interventions for neurodivergent children in South Asian contexts, demonstrating improvements in parental knowledge, parent–child interaction, and child developmental outcomes [13,14]. However, these reviews have focused on condition-specific interventions rather than general parenting interventions. Moreover, one recent review noted the lack of rigorous evaluation of how parenting interventions are culturally adapted for South Asian families, emphasizing that cultural adaptation processes and their impact on outcomes remain underexplored [15]. In South Asia, several parenting interventions have been culturally adapted and evaluated to support child development and parental well-being, incorporating home-based delivery, traditional games and songs, and culturally relevant materials [16,17,18].

To date, no systematic review has evaluated parenting interventions specifically targeting parents of young children within the South Asian context, nor examined the effectiveness of these interventions in relation to their level of cultural adaptation. Understanding the effectiveness of parenting interventions in South Asian populations, as well as the level of cultural adaptation necessary to optimize their effectiveness, is essential. This study contributes to the Sustainable Development Goals (SDGs) [19], particularly SDG 3 (Good Health and Well-Being), by addressing parental mental health and, consequently, child development. It also contributes to SDG 10 (Reduced Inequalities) by emphasizing the importance of cultural relevance in the development of parenting interventions for South Asian populations.

The aim of this systematic review is to assess the effectiveness of parenting interventions on children’s developmental outcomes and parents’ outcomes related to parenting and well-being. To address gaps in understanding the extent of cultural adaptation required to optimize effectiveness for South Asian populations, this review applies Bernal’s Ecological Validity Model to systematically assess cultural adaptation processes and components within parenting interventions, and examines whether the quality of adaptation is associated with intervention efficacy [20].

## 2. Methods

### 2.1. Eligibility Criteria

#### 2.1.1. Population

The studies inclusive of parents (both genders) of children aged between 0 and 12 years old from South Asia or a South Asian ethnic minority group in a non-South Asian country were included. The South Asian bracket included participants from Pakistan, India, Nepal, Bhutan, Bangladesh, Maldives, and Sri Lanka and ethnic minorities from South Asia living in high income countries (HICS). By ethnic minorities we mean a group of people who share a common culture, religion, language, or nationality [21]. We focused on ethnic groups that are minority groups in the country in which the intervention was delivered. For studies with mixed samples, 50% of the sample must be constituted of South Asian population.

#### 2.1.2. Intervention

Interventions aimed at enhancing supportive parenting through psychoeducational programs and/or training for parents of young children were included. Comparators included usual care, other active non-parenting interventions, or no intervention.

#### 2.1.3. Outcome

Outcomes measures included at least one of the following: (1) improvement in parenting (reduction in harsh parenting as well as improvement in parenting including parenting knowledge, involvement); (2) parents health (physical and mental health); and (3) child outcomes (cognitive, physical, and behavioral outcomes).

#### 2.1.4. Design

Any quantitative studies (RCTs, quasi-randomized and single-arm trials) based on testing parenting interventions for South Asians or qualitative studies (mixed-methods studies, case studies, grounded theory, and ethnographic methods) exploring participant’s experiences parenting intervention was included.

All papers published in peer-reviewed journals or as dissertations in English, or with an English translation available if published in another language, were included. We excluded interventions targeting children with learning difficulties, disabilities, or severe mental health diagnoses.

### 2.2. Information Sources and Search Strategy

We searched six electronic databases (CINAHL, MEDLINE, Science Direct, PsychINFO, PubMed, and Cochrane library) for original studies from January 1806 to August 2023. The reference lists of the included studies were searched to identify any additional eligible studies that might not have been picked by the searches. If necessary, study authors were contacted to obtain further information on the intervention and inclusion criteria.

**Figure 1:**
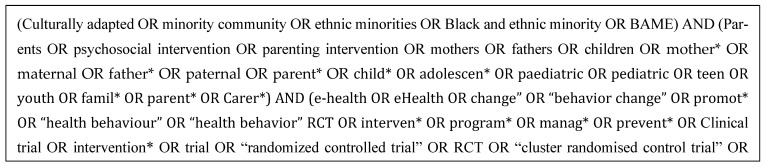
Search terms used for the databases.

The following search strategy was used for the databases to identify relevant studies. The search strategy was manually developed by AS and UA, and was adapted for each database to account for differences in indexing and controlled vocabulary (e.g., MeSH terms), with appropriate keywords used where controlled terms were not available (Figure 1).

### 2.3. Data Selection and Data Collection Process

Covidence [22] was used to facilitate screening after deduplication. Screening was conducted in two stages: first, titles and abstracts were screened using a set of inclusion criteria, and then the full texts of studies that met eligibility at the title/abstract stage were retrieved. Both screening stages and selection of studies were completed independently by two reviewers (AS and UA), and any disagreements were resolved through a consensus meeting with a third reviewer (KL).

A pre-piloted Excel data extraction form was used to collect data. We extracted information on descriptive study characteristics, including demographic data, country, methodology, number of participants, type of intervention, parent characteristics, and outcomes (e.g., age, gender, mental health outcomes, and parenting outcomes), as well as child characteristics and outcomes (e.g., age, behavioral and developmental outcomes). Two reviewers (AS and UA) extracted the data independently, and a third reviewer (KL) checked 3 of the 17 studies to ensure consistency and clarity.

### 2.4. Data Quality, Reflexivity and Reviewer Positionality

In accordance with PRISMA 2020 guidance, data quality and reflexivity were ensured through independent study selection, risk of bias assessment, and grading of cultural adaptation for each included study by two reviewers (AS and UA), with discrepancies resolved through discussion or adjudication by a third reviewer (KL).

### 2.5. Protocol and Registration

This systematic review adheres to the PRISMA-P (Preferred Reporting Items for Systematic Review and Meta-Analysis Protocols) checklist [23] and is reported according to PRISMA and Cochrane systematic review guidelines. Prospero registration number: CRD42022361920.

### 2.6. Risk of Bias Assessment and Assessment of the Certainty of the Evidence

All cluster and individually randomized controlled trials were appraised using the Revised Cochrane Risk of Bias Tool for Randomized Trials (RoB 2.0) [24]. Each trial was evaluated across key domains, including the randomization process, allocation concealment, deviations from intended interventions, attrition, and outcome reporting, and was subsequently classified as low risk, some concerns, or high risk of bias [25].

Qualitative studies were assessed using the Critical Appraisal Skills Program (CASP) checklist [26,27]. All eligible studies were appraised independently by two reviewers (AS, UA), with discrepancies resolved through discussion with a third reviewer (KL).

The overall certainty of the evidence for quantitative outcomes was evaluated using the GRADE approach, taking into account risk of bias, inconsistency, indirectness, imprecision, and publication bias.

### 2.7. Assessment of Cultural Adaptation

AS and UA independently reviewed each study for cultural adaptation and rated them using Bernal’s Ecological Validity Model (EVM) [20], which has a strong theoretical foundation and is widely used in cross-cultural psychosocial and behavioral intervention research [28]. In consideration for the current review where parenting interventions were assessed for sensitivity to cultural norms and family practices, the EVM provided an appropriate multidimensional framework, enabling in-depth evaluation of cultural adaptation across eight domains (language, persons, metaphors, content, concepts, goals, methods, and context). This allowed assessment beyond surface-level adaptations such as translation [29].

The EVM model was applied and cross-checked across all 8 domains; study authors were contacted for more information when clarification was needed. Following a consensus between AS, UA, MP, and AH, studies meeting seven or more EVM domains were classified as demonstrating satisfactory cultural adaptation.

### 2.8. Data Analysis

Meta-analysis was conducted on all the studies reporting amenable data on children’s outcomes, parental mental health outcomes, and parenting outcomes. Meta-analyses were conducted in Stata 16 with the metaan command using random-effect models to account for anticipated heterogeneity [30]. Between-study heterogeneity was assessed using the I^2^ statistic. Sensitivity analyses were conducted [31], in which studies with high risk of bias and with an insufficient cultural adaptation process were excluded from the analysis. In analyses that included 10 or more studies, we inspected the funnel plots (using the meta funnel command) [32] and conducted the Egger test (using the meta bias command) to assess for small-sample bias [33]. For cluster randomized clinical trials, the precision of analyses was adjusted using a sample size/variation inflation method, assuming an intraclass correlation of 0.02. We also applied grade approach to overall certainty of evidence.

Quantitative studies that did not provide data suitable for meta-analysis were synthesized narratively and interpreted in the context of the meta-analytic findings. Qualitative studies were synthesized using manual inductive thematic analysis, following Braun and Clarke’s framework for qualitative synthesis [34]. All relevant data on participants’ feedback regarding the culturally adapted parenting interventions, engagement, and perceived impact were extracted verbatim from the results sections of the included studies. The first author (AS) organized the data in an Excel table and generated initial codes from the extracted information. Similar codes were then grouped into themes, which were reviewed and refined in collaboration with UA and KL based on their relevance to the review inclusion criteria. To ensure consistency, themes were cross-checked between AS and UA, with any disagreements resolved by KL. Final themes were agreed upon by all authors.

## 3. Results

**Figure 2:**
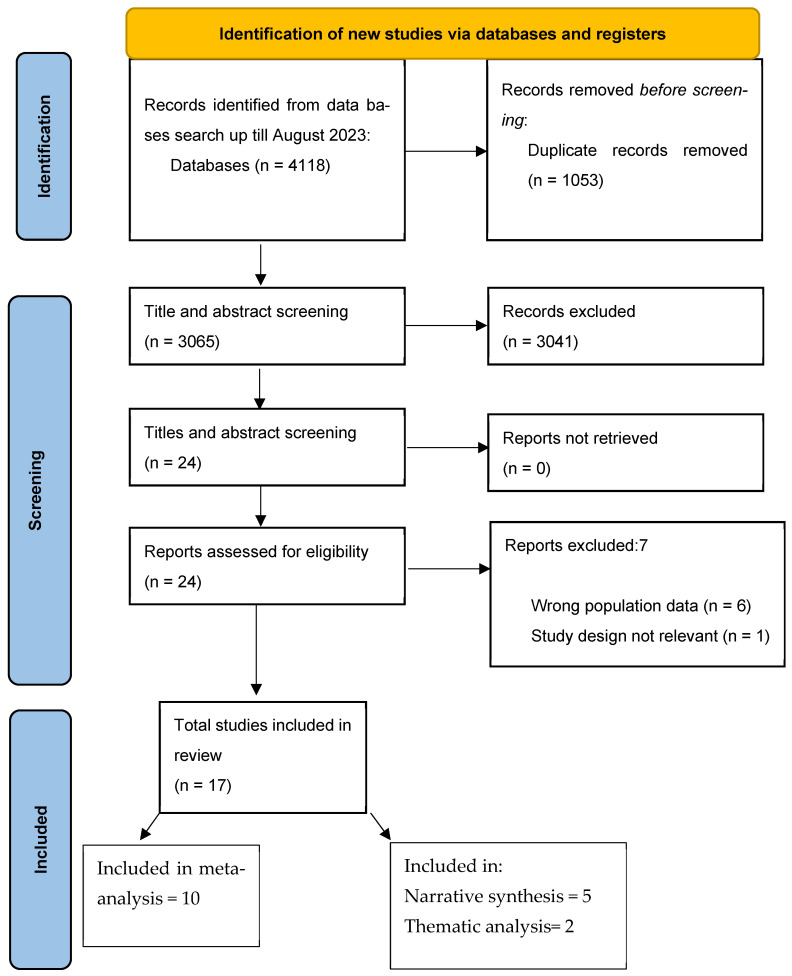
PRISMA flow diagram for literature search.

The literature search flow diagram is presented in Figure 2. A total of 4118 articles were identified through the searches. After removing duplicates, 3065 studies were screened based on titles and abstracts, resulting in the exclusion of 3041 studies and leaving 24 for full-text screening. Following full-text review, 7 studies were excluded for not meeting the eligibility criteria. In total, 17 studies were included in the review, comprising 15 quantitative intervention studies and 2 linked qualitative studies. Of the 15 quantitative studies, 10 were included in the meta-analysis, while the remaining 5 were synthesized narratively. The two qualitative studies were analyzed using a thematic analysis framework.

The key findings relating to parenting knowledge, child cognitive development, and parental depression are informed by the quantitative synthesis and are closely linked to the methodological characteristics of the included studies. While sensitivity analyses were conducted where sufficient data were available, it was not possible to perform sensitivity analyses across all outcome domains due to the limited number of studies reporting specific outcomes.

In several analyses, particularly those relating to parental mental health and child developmental outcomes, only a small number of studies met the inclusion criteria, which limited the ability to further stratify results based on methodological quality or level of cultural adaptation.

### 3.1. Study Characteristics

Most of the studies were completed in Pakistan (8) and 2 papers were published from a single study conducted in Norway on the Pakistani population, followed by 4 studies in Bangladesh and 3 in India. The total population reported in the studies was 8088 inclusive of mothers, fathers, children, and community workers. One study reported findings for 18 fathers and the remaining 8070 of the population were mothers or mother-and-children dyads. The two qualitative studies recruited 26 participants altogether: 24 were mothers and 2 participants were community health workers. All the parents’ ages reported varied between 23 years and 36 years, and the children’s ages reported were from 0 to 6 years. Out of 17, 15 of the studies’ age ranges for children varied between 0 and 3 years. Out of 15 quantitative studies, 9 studies used cluster randomized controlled trials, 5 randomized controlled trials, and 1 pre–post design. The two qualitative studies used semi-structured interviews.

### 3.2. Intervention and Outcome Characteristics

Overall, seven types of parenting interventions were used across the studies. Among the reviewed interventions, psychosocial stimulation was the most frequently adopted approach followed by learning through play plus a thinking health program [16,17,35,36]. One study used learning through play [37]. The remaining five studies used a combined educational intervention, parent management training—Oregan model [38,39], parenting program [18], and Reach-Up curriculum for early childhood stimulation [40].

The delivery mode varied between group sessions, individually delivered sessions, and mix of individual and group sessions. The mixed delivery method [18,37,38,40,41,42,43] was used by six studies. The remaining four studies used a one-to-one delivery method [44,45,46,47]. The length of intervention varied between 24 months and 12 weeks.

Alongside parenting intervention, ten studies included other interventions such as nutritional education [42,45,46], breastfeeding or infant feeding counselling [44,47], and a thinking healthy program [16,17,35,36,48].

For the review, only the results from the parenting interventions designed to improve mother’s and children’s health and parenting will be discussed. Details of each intervention are provided in Supplementary Material.

A summary of all reported outcomes, including parenting, parental health, and child outcomes, is presented in Table 1 for clarity and ease of reference. Summary of outcomes from included studies is presented in Table 2.

### 3.3. Results of Risk of Bias Assessment

Among the trials included in this study, 3 had a low risk of bias, 3 had some concerns, and the remaining 8 were rated as high risk of bias.

For the two qualitative studies, one is rated as having a low risk of bias, meeting 9 out of 10 questions on the CASP checklist [48]. The second study [40] is rated as having a moderate risk of bias, meeting 7 out of 10 questions on the CASP checklist.

### 3.4. Results of Cultural Adaptation

Of the 17 studies, 5 met all 8 components of Berlin’s cultural adaptation requirements, 2 met 7 components, 1 study met 6 components, 3 met 5 components, 2 met 4 components, 2 met 3 components, and 2 failed to meet any of the requirements. More information about the cultural adaptation is in Supplementary Material.

### 3.5. Meta-Analysis of Parenting Outcomes

Overall, meta-analysis was performed on ten studies: [17,18,35,36,37,41,42,43,45,47].

Parental interventions showed moderate significant improvements in parenting knowledge in comparison to treatment as usual (SMD = 0.51 95% CI = 0.25, 0.76; k = 5) but heterogeneity was high (I^2^ = 96%, *p* < 0.00).

Sensitivity analyses in which studies with low risk of bias and satisfactory level of cultural adaptation were retained respectively, showing that parental interventions were associated with moderate to high improvements in parental knowledge (SMD = 0.66 CI = 0.33, 0.99; k = 2), with moderate to high heterogeneity (I^2^ = 76.6% *p* = 0.014) for low risk of bias studies; [18,41] and (SMD = 0.69 CI = 0.49,0.89; I^2^ = 71.0% *p* = 0.008, k = 4) for studies with satisfactory level of cultural adaptation.

Parental interventions were associated with small significant improvements in parental involvement in comparison to usual care (SMD = 0.36 CI = 0.00,0.72, k = 3) in the presence of high heterogeneity (I^2^ = 96%, *p* < 0.00).

### 3.6. Meta-Analysis of Parental Health Outcomes

Compared to usual care, parental interventions were associated with significant reductions in symptoms of depression among parents (SMD= −0.77 CI = −1.20−0.34; I^2^ = 93%, *p* < 0.00, k = 4); there were moderate significant improvements in parental self-esteem (SMD = 0.56 CI = 0.26 -1.06; I^2^ = 85%, *p* < 0.01, k = 2) and high significant reductions in EQ5D disability symptoms (SMD 0.82 CI = 0.68- 0.96; I^2^ = 0.0% *p* < 0.465, k = 2). As indicated by the I^2^ statistics, most of these analyses included moderate to high heterogeneity.

Parental interventions were associated with low significant changes in reducing post-natal depression (SMD = 0.15 CI = −1.00-1.30; I^2^ = 98%, *p* < 0.00, k = 3) [17,35,36] and physical QoL disability symptoms (SMD = 0.27 CI = −1.22,1.75; I^2^ = 98.9%, *p* < 0.00, k = 2) compared to usual care.

### 3.7. Meta-Analysis of Child Outcomes

Parental interventions were associated with high significant improvements in children’s cognition in comparison to usual care (SMD = 0.84, 95 CI = 0.32 to 1.36. However, heterogeneity was high (I^2^ = 98.3%, *p* < 0.00, k = 6).

A sensitivity analysis based on risk of bias showed a consistent effect size with the main analysis (SMD = 0.79 CI = 0.17 to 1.41; I^2^ = 96.0%, *p* = 0.000, k = 3) [18,41,47].

A further sensitivity analysis in which only studies with sufficient level of cultural adaptation were retained showed that parenting interventions were associated with high significant improvements in child cognitive outcomes (SMD = 1.48 CI = 0.52, 2.45); I^2^ = 98.1% *p* = 0.000) [17,18,41]. Although the difference was non-significant, it appears that interventions with sufficient level of cultural adaptation were associated with greater improvements in the child cognitive outcomes than studies with insufficient level of cultural adaptation.

Compared to usual care, parental interventions were associated with high significant improvements in children’s language development (SMD = 0.79 CI = 0.25-1.33; I^2^ = 98.3%, *p* < 0.00, k = 4) [17,18,42,45] and moderate significant improvement in social development (SMD = 0.54 CI = 0.16-0.91; I^2^ = 97%, *p* < 0.00, k = 4) [17,18,42,45]. Parental interventions were not associated with significant improvements in children’s emotional development (SMD = 0.05 CI = −0.09-0.19; I^2^ = 35.9%, *p* < 0.21, k = 2) [41,42] and motor development (SMD = 0.50 CI = −0.09-1.08; I^2^ = 99%, *p* < 0.00, k = 5) [17,41,42,45,47] compared to usual care. Across most of these analyses, heterogeneity was substantial as indicated by the I^2^ statistic.

### 3.8. Results of the Certainty of the Evidence (GRADE Assessment)

Culturally adapted parenting interventions show promising effects on parental and child outcomes. However, the overall certainty of evidence is low due to methodological limitations, high heterogeneity across studies, and imprecision from small sample sizes or wide confidence intervals. Emotional and motor development outcomes showed little or no clear benefit, adding to the uncertainty.

### 3.9. Narrative Summary of Quantitative Studies with Non-Amenable Data for Meta-Analysis

Narrative synthesis of five quantitative studies that reported non-amenable data for meta-analysis further supported the main meta-analysis findings above. Parenting interventions led to a reduction in fathers’ depression and parenting stress in a pre–post intervention assessment at six months follow-up [16], as well as a reduction in harsh parenting in mothers [39,49]. Again, effects were seen for parenting interventions on improvement in knowledge of developmental milestones and maternal distress [39].

In terms of child outcomes, evidence was found for significant intervention improvements in cognition [46], language [46], and communication, while there was no effect found on motor skills [44].

### 3.10. Thematic Synthesis of Qualitative Studies

The thematic analysis identified three overarching themes associated with parenting intervention outcomes: (1) maternal empowerment and positive role transformation; (2) improvements in maternal mental health; and (3) factors influencing engagement and participation.

Theme 1: Mother’s empowerment and positive role transformation.

Both qualitative studies found that the mothers appeared more confident and able to deal with difficult situations and reported greater outward thinking than inwards [40,48]. They demonstrated positive nurturing roles and appeared to be more interactive and empowered [48].

“I dealt with my children harshly, but after attending the intervention I learned how to spend time with them and understand them”.

(p 7)

“Now I try to problem solve myself”.

(p 5)

Theme 2: Improved Mental Health outcomes.

Mothers reported focusing more on their health including using behavioral activation to reduce anxiety, eating healthy, and giving importance to child-stimulating activities [40].

“Now I do not get obsessed with worries like I did before”.

(p 3)

Theme 3: Engagement factors.

The qualitative studies also provided some evidence on factors that may influence participation and engagement with the interventions. Mothers felt assistance with house chores (from family members) enabled them to attend the sessions. The women often felt uncomfortable discussing sensitive issues, and occasional engagement of husbands and extended family would put them at ease as gate keepers [40]. One study reported the female facilitator as the catalyst to success in engaging the participants. They felt intervention sessions gave them a rare opportunity of being able to share their difficulties and gaining the adequate empathy and support.

“ If I could not finish my work, and the session is about to begin, my mother-in-law does the remaining work and let me go for the session”.

(IDI-6)

## 4. Discussion

### 4.1. Summary of the Findings and Implications

Culturally adapted parenting interventions appear to improve parents’ functional capacity and psychological wellbeing, as indicated by reductions in disability symptoms and depressive symptoms. Qualitative data suggest that these interventions may enhance emotional support and confidence in parenting, which could help explain these benefits. However, the lack of significant improvements in post-natal depression highlights that parenting interventions alone may not sufficiently address postpartum-specific mental health needs. These mixed findings are consistent with broader evidence from the global literature, where the impact of parenting interventions on parental stress and depression is variable. While some studies report limited impact of parenting interventions on parental stress and depression [12,50,51], others demonstrate moderate improvements [10,52]. Studies that combine psychosocial support with parenting programs tend to show greater improvements, suggesting that integrated approaches may be more effective. This aligns with evidence that parental depression can reduce engagement with interventions [53,54], indicating that targeted mental health components may be necessary to optimize outcomes, particularly for postpartum mothers.

Culturally adapted parenting interventions appear to have a positive, albeit modest, impact on parental involvement and a clearer effect on parenting knowledge. The observed small improvements in involvement may reflect cultural and contextual factors, such as joint-family systems and collectivist practices, where caregiving responsibilities are distributed across multiple family members [55]. This suggests that engaging the broader family in interventions could enhance effectiveness and support deeper cultural alignment. In contrast, improvements in parenting knowledge were more pronounced, particularly in studies that implemented deeper cultural adaptations. Interventions like LTP-Plus [17], which incorporated locally meaningful examples and linked play activities to culturally familiar experiences, appear to have strengthened parents’ understanding and application of play-based learning. Qualitative findings further indicate that increased knowledge translated into reductions in harsh parenting and more positive parent–child interactions, highlighting the importance of culturally embedded strategies for enhancing the quality of parenting practices.

Culturally adapted parenting interventions appear to substantially benefit children’s cognitive and language development, particularly when interventions incorporate deeper cultural adaptations. Studies that met a greater number of Bernal’s Ecological Validity Model components tended to show stronger effects, suggesting that carefully designed, culturally congruent strategies can enhance engagement and learning. For example, integrating traditional games and songs familiar to families and using pictorial materials adapted to local literacy levels likely supported parental understanding and participation [18]. Qualitative findings further indicate that family support and culturally aligned facilitators were important in sustaining engagement, highlighting how structural and contextual adaptations may contribute to improved developmental outcomes for children.

Overall, this review aligns with prior evidence emphasizing the benefits of culturally adapted interventions for South Asian families, particularly in enhancing children’s cognitive development and parenting knowledge [12,56,57]. While cultural adaptation is increasingly recognized as important globally, not all interventions follow a rigorous, systematic approach [15]. This review reinforces the need for modifications that go beyond surface-level changes, such as simple language translation, to achieve meaningful and sustained intervention outcomes [58,59].

### 4.2. Strengths and Limitations

This systematic review is among the first to comprehensively examine the effects of culturally adapted parenting interventions on multiple outcomes within the South Asian community, including parenting practices, child development, and parental health. A key strength of the review is its focus on cultural adaptation, providing unique insights and practical guidance for future work. This emphasis on cultural specificity is essential for developing effective, culturally sensitive interventions. The review employed a diverse range of methodological approaches, including meta-analysis and narrative synthesis of both quantitative and qualitative data, as recommended [60]. Such methodological diversity is crucial for understanding complex interventions.

However, several limitations should be acknowledged. The high risk of bias in the quantitative studies and the substantial heterogeneity observed in the meta-analyses pose challenges for drawing definitive conclusions. This heterogeneity was particularly evident in the types of interventions, the dosage of parenting components within mixed interventions, and the length of delivery, creating gaps in identifying the optimal intervention for this cultural group. Additionally, the sample largely consisted of children under three years of age, limiting the generalizability of findings to older children. Further research is therefore needed to explore the effects of parenting interventions on children over three years. The inclusion of comorbid intervention components in some studies also adds complexity to interpreting the results. Overall, the GRADE assessment indicated a low certainty of evidence. Moreover, the limited number of qualitative studies restricts understanding of certain outcomes and the broader contextual factors influencing intervention effectiveness. While we conducted sensitivity analyses where possible, some outcomes included a small number of studies, restricting further exploration of methodological or cultural adaptation effects.

Despite these limitations, this review’s focus on cultural adaptation in parenting interventions provides valuable insights and underscores the importance of culturally tailored approaches for enhancing parenting practices and child development outcomes.

## 5. Conclusions

Findings from this systematic review suggest that culturally adapted parenting interventions show promising effects on key parenting and child developmental outcomes among South Asian families. These results highlight the importance of situating interventions within culturally relevant contexts and support global policy calls for comprehensive, context-specific, and culturally responsive parenting programs [61].

However, variability in outcomes particularly in emotional and motor development, parental depression, and stress indicates that intervention effects are not uniform across all domains. The limited number of qualitative studies also constrains understanding of the mechanisms and contextual factors underlying intervention effectiveness. This underscores the need for future research to explore targeted adaptations, including the integration of psychosocial support components, to enhance outcomes for both parents and children.

Overall, given the low certainty of evidence, there is a clear need for more rigorous trials with consistent reporting of parenting-related outcomes, alongside detailed documentation of intervention components, methodological quality, and cultural adaptation processes. Such research will help clarify which cultural adaptations and parenting interventions are most effective for South Asian populations.

## Figures and Tables

**Table 1:** Characteristics of included studies.

Author	Study Type	Population	Intervention	Sample Size	Mothers Age	Children’s Age	Samples Characteristics	Parenting Outcomes	Child Outcomes	Parent’s Health Outcomes
Jena D. Hamadani (2006) [41]	Cluster randomized trial	Bangladesh	Psychosocial Stimulation	299	Not mentioned	Mean age = 14.6 months	Undernourished children	Yes	Yes	No
Sally Grantham-McGregor (2020) [42]	Cluster randomized controlled trial	India	Psychosocial Stimulation	1449	Not mentioned	Mean age = 7–16 months	Rural population	No	Yes	Yes
Gulshan Ara (2019) [44]	Cluster randomized controlled trial	Bangladesh	Peer counselling	378	Mean age = 23.6	Mean ag e = not reported.	Mothers living in Urban slums	No	Yes	Yes
Aisha K. Yousafzai (2015) [43]	A cluster randomized factorial effectiveness	Pakistan	Responsive stimulation intervention	1489	1489	Mean age = not reported	Mothers living in impoverished communities	Yes	No	Yes
Nusrat Husain (2017) [36]	Rater blind randomized controlled trial with two parallel groups	Pakistan	Learning through play + thinking healthy program.	247	Mean age = 28.2	Mean age = 28.2	Women with symptoms of maternal depression	Yes	No	Yes
Nusrat Husain (2021) [35]	Randomized controlled trial	Pakistan	Learning through play + thinking healthy program	107	Mean age = 27	Mean age = 14.1l	Women with symptoms of maternal depression	Yes	No	Yes
Aisha K. Yousafzai (2014) [45]	Cluster randomized study 2 ×2 factorial design	Pakistan	Responsive stimulation group	1489	Age = not mentioned	Age range = 2.5 months	Mothers in underserved areas	No	Yes	No
Alison Andrew (2020) [46]	Cluster randomized trial	India	Psycho-stimulation intervention	421		Mean age = 14.9	Mothers in slums	No	Yes	Yes
Shahnaz Vazir (2013) [47]	Cluster randomized trial	India	A combined educational intervention (responsive complementary feeding and mother– child interaction); (RCFG)	600	Age = not mentioned	Age range = 3–15 months.	Mother-and-child dyads recruited from rural area	No	Yes	Yes
Ragnhild Bjorknes (2015) [39]	Randomized control trial	Norway (Pakistani immigrants)	Parent management training—Oregan model (PMTO)	96	Mean age = 33.6	Mean age = 5.9	Pakistani immigrant mothers experiencing mental distress	Yes	No	No
Frances E. Aboud (2013) [18]	Cluster field trial	Bangladesh	Parenting program	463	Not mentioned	Age range = 4–14 months	Mothers in a community cohort	Yes	Yes	Yes
Muhammed I. Husain (2021) [16]	Pre–post design	Pakistan	Learning through play Dads:	18	Fathers mean age = 33	Age range = 0–3 Years	Fathers experiencing paternal depression	Yes	No	Yes
Nusrat Husain (2021) [17]	Randomized controlled trial.	Pakistan	Learning through Play Plus (LTP+)	774	Age = not mentioned	Age range = 0–30 months	Mothers with maternal depression	Yes	Yes	Yes
A Rahman (2009) [37]	Cluster randomized design.	Pakistan	Learning through play	162	Age = 17–44	Age range = 0–36 months	Mothers in a community in rural settings	Yes	No	Yes
Ragnhild Bjorknes (2013) [49]	Randomized controlled trial.	Norway (Pakistani immigrants)	Parent management training—Oregan model (PMTO)	96	Mean Age = 33.71	Mean age = 5.90	Mothers experiencing maternal distress	Yes	No	No
Fahmida Akter (2020) [40]	Semi-structured interviews	Bangladesh	Reach up curriculum for early childhood.	10	Age = not mentioned	Age range = 0–24 months	Pregnant and lactating mothers	Yes	No	Yes
Nusrat Husain (2017) [48]	Semi-structured interviews	Pakistan	Learning through play +	8	Mean age = 36.4	Mean age = 0.30	Mothers experiencing maternal depression	Yes	No	Yes

**Table 2:** Summary of outcomes from included studies.

Outcome Domain	Specific Outcome	Type of Analysis	
Parenting	Parenting knowledge	Meta-analysis	SMD = 0.51, 95% CI = 0.25–0.76; I^2^ = 96%, k = 5
	Parental involvement	Meta-analysis	SMD = 0.36, 95% CI = 0.00–0.72; I^2^ = 96%, k = 3
	Reduction in harsh parenting	Narrative synthesis	Pre-post reduction in mothers’ harsh parenting, k = 3
Parent Health	Depression	Meta-analysis	SMD = −0.77, 95% CI = −1.20 to −0.34; I^2^ = 93%, k = 4
	Maternal distress	Narrative synthesis	Reduction in maternal distress reported, k = 2
	Self-esteem	Meta-analysis	SMD = 0.56, 95% CI = 0.26–1.06; I^2^ = 85%, k = 2
	Post-natal depression	Meta-analysis	SMD = 0.15, 95% CI = −1.00 to 1.30; I^2^ = 98%, k = 3
	Physical QoL/disability	Meta-analysis	SMD = 0.82 (EQ5D), 95% CI = 0.68–0.96; I^2^ = 0%, k = 2
Child Outcomes	Cognition	Meta-analysis	SMD = 0.84, 95% CI = 0.32–1.36; I^2^ = 98%, k = 6
	Language development	Meta-analysis	SMD = 0.79, 95% CI = 0.25–1.33; I^2^ = 98%, k = 4
	Social development	Meta-analysis	SMD = 0.54, 95% CI = 0.16–0.91; I^2^ = 97%, k = 4
	Emotional development	Meta-analysis	SMD = 0.05, 95% CI = −0.09–0.19; I^2^ = 36%, k = 2
	Motor development	Meta-analysis	SMD = 0.50, 95% CI = −0.09–1.08; I^2^ = 99%, k = 5
Qualitative/Thematic	Maternal empowerment & positive role transformation	Thematic synthesis	Mothers reported greater confidence, positive nurturing roles, and interactive engagement, k = 2
	Improvements in maternal mental health	Thematic synthesis	Behavioral activation, reduced anxiety, improved child-focused activities, k = 2
	Factors influencing engagement	Thematic synthesis	Family support, female facilitators, cultural considerations, k = 2

## Data Availability

Data can be made available upon request. The data are not publicly available due to their large size.

## Supplementary Materials

The following supporting information can be downloaded at: https://www.mdpi.com/article/10.3390/children13010086/s1.
