# Supplementary material for: Effectiveness and Cultural Adaptation of Parenting Interventions for South Asian Families: A Mixed-Methods Systematic Review Using Bernal’s Ecological Validity Model
Source: Children (Basel). 2026 Jan 6;13(1):86. doi: 10.3390/children13010086 (PMC12839602; doi:10.3390/children13010086)
Supplement: Supplementary file 1 [file children-13-00086-s001.zip › children-4024417-supplementary.pdf]

LIST OF Supplementary Materials

Supplementary Material S1:

The extract below illustrates search terms that will be used to identify research studies of culturally adapted intervention parenting interventions for South Asians:

(Culturally adapted OR minority community OR ethnic minorities OR Black and ethnic minority OR BAME) AND (Parents OR psychosocial intervention OR parenting intervention OR mothers OR fathers OR children OR mother* OR maternal OR father* OR paternal OR parent* OR child* OR adolescen* OR paediatric OR pediatric OR teen OR youth OR famil* OR parent* OR Carer*) AND (e-health OR eHealth OR change" OR "behavior change" OR promot* OR "health behaviour" OR "health behavior" RCT OR interven* OR program* OR manag* OR prevent* OR Clinical trial OR intervention* OR trial OR "randomized controlled trial" OR RCT OR "cluster randomised control trial" OR "cluster randomized control trial" OR "crossover" OR Feasibility OR Pilot OR program evaluation OR acceptability OR sustainability OR qualitative) AND (Pakistan* OR India* OR Nepal* OR Bhutan* OR Bangladesh* OR Maldiv* OR Sri Lanka*)

Supplementary Material S2: *List of parenting intervention included in the studies.*

PARENTING INTERVENTION	INTERVENTION DESCRIPTION	STUDIES
Psychosocial stimulation.	Improving the mother-child interaction, providing developmentally appropriate activities. The importance of praising children, giving positive feedback, chatting with them, punishment was discouraged. Included traditional games and songs and produced low-cost picture books suitable for Bangladeshi children and mothers with little reading ability.	(1-3) (4-7)
Learning through play (1)	Provide parents with developmentally appropriate messages to stimulate early child development in a culturally appropriate format. Participant's were given a pictorial calendar devised for parents which depicts eight	(7, 8)

	successive stages of child development from birth to 3 years along with illustrations of parent-child play and other activities that promote parental involvement, learning, and attachment.	
Learning through play + Thinking healthy programme (THP)	In addition to LTP intervention, the THP manual includes step-by-step instructions and uses CBT techniques. Each session organized into five modules. Each module focuses on the following three areas: the mother's personal health, the mother- infant relationship, and the psychosocial support of significant others.	(9-12)
Combined educational intervention.	The participants were provided eight messages and skills on responsive feeding, developmental stimulation messages using five simple toys. - age-appropriate messages and skills on how to understand and respond to infants' cues of hunger/appetite or satiation.<br>Received developmentally appropriate toys five times during the intervention with instructions on how to use them to engage and play with their children.	(13)
Parent management training-Oregan model (PMTO)	The intervention effectively prevents and improves children's behavior problems by replacing coercive interactions with positive parenting practices. The focus is on teaching parents skills to prevent and children's conduct problems (e.g., aggression, externalising, antisocial, delinquent behavior) as well as improve prosocial behavior (e.g., social skills, academic functioning).	(14, 15)
Parenting programme.	The program concerned parenting practices related to health, nutrition, communication and play: specifically showing love and avoiding harsh discipline and responsive	(16)

	self-feeding. -government- paid family welfare assistants who were instructed to deliver messages during a 10-min counselling session to mothers of young children at home and at their community clinics	
Reach-Up curriculum for early childhood stimulation	Components to improve Psychosocial stimulation (e.g. teach mothers on age appropriate activities, home made books and interaction with children during daily household).<br>Health care and nutrition (e.g. Teach mothers on importance of colostrum, breastfeeding, pregnancy and delivery problems)<br>Maternal mental health. (e.g. behavioural activation through thinking healthy program on managing relations with child and people around her.	(17)

Supplementary Material S3: *Details of measures used for child development, Parenting and parent's health measures in all the studies.*

MEASURES FOR CHILD DEVELOPMENT

**Measures**	**Studies**
Revised version of Bayley Scales of Infant Development (BSID-II)	(1, 13, 18)
Bayley scales of infant and toddler development scale	(3, 4, 6, 16)
Anthropometer including behaviour 9- point scale	(1)
World Health Organisation (WHO) indicators of infant and young child feeding practices.	(3, 13)
The Ages and stages 3rd edition questionnaire	(6, 11, 18)
ECD module of the UNICEF Multiple Cluster Index Surveys	(4)
Infant Development Questionnaire (IDQ)	(8, 9)

Conduct problem composite (CP composite)	(14, 15)
Home inventory measure	(16)
Infant Development Questionnaire (IDQ)	(8)
Teacher report form (TRF)	(15)
Social skills rating system	(15)

MEASURES TO ASSESS PARENTING:

**Measures**	**Studies**
The learning through, Attitude and practices (KAP)	(7, 11)
Observation of Mother and Child Interaction (OMCI)	(4)
Maternal Attachment Inventory (MAI)	(10)
UNICEF's Family Care Indicators (FCI)	(6)
Parent Practices Interview	(14, 15)
Home Observation for Measurement of the Environment.	(11)
Maternal knowledge of child development	(6)
Mothers' knowledge of the ages for child development	(16)
Maternal knowledge & belief	(13)
Family satisfaction Survey (FFS;)	(15)

MEASURES TO ASSESS PARENT'S DISTRESS:

**Measures**	**Studies**
Edinburgh Postnatal Depression Scale (EPDS)	(7, 9, 10)
The Hamilton Depression Rating Scale (HAM-D)	(7, 9, 10)
Short versions of the Center for Epidemiologic Studies depression scale (CES-D):	(6, 13, 16)
Self-reporting questionnaire (SRQ-20)	(8, 9)

Short version of the Hopkins Symptom Check List-5 (SCL-5)	(14)
Parenting Stress Index – Short Form (PSI)	(7)
Rosenberg Self-Esteem Scale (Rosen- berg SES)	(7, 9)
The Multidimensional Scale of Perceived Social (MPSS)	(7, 9, 11)
Social Support Scale (OSLO-3)	(6)
The Brief Disability Questionnaire (BDQ)	(7, 9, 11)
Euro-QoL (EQ-5D)	(7, 10)
The Patient Health Questionnaire (PHQ-9)	(11)

<u>Supplementary Material S4</u>: <u>Results of the risk of bias assessment for RCTs</u>

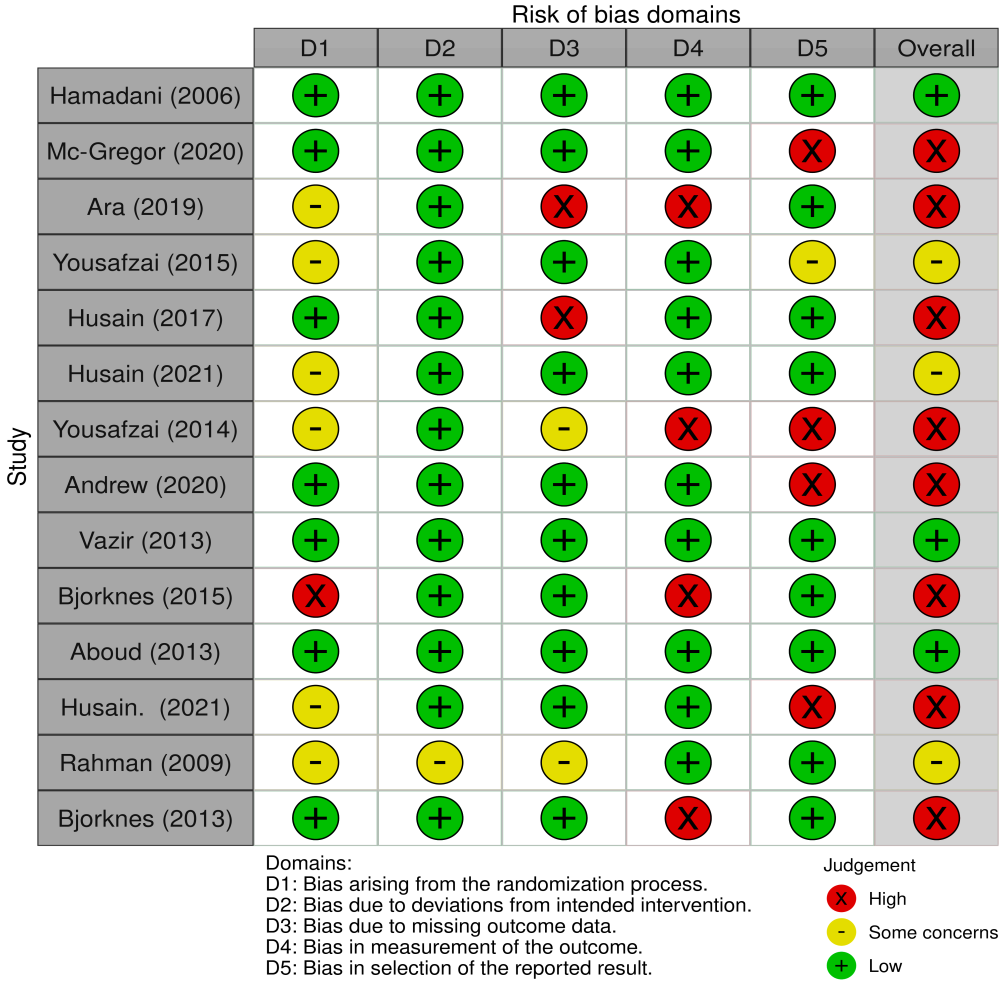

Supplementary Material S5: *Summary of studies meeting cultural adaptation criteria*

Ecological validity model component	Total number of studies meeting the criteria	Studies meeting the criteria.	Examples of studies meeting the criteria.
Language	15	(1, 3, 7-19)	Local language considered in delivery of the intervention.
Persons	15	(1, 3, 6-8, 15, 17, 18).	Therapist and clients were ethnically matched, e.g. not just matched on language but recruited from same village as the participant's.
Metaphors	9	(1, 7-12, 16, 17)	Cultural uniqueness concepts such as joint family system in south Asian culture were addressed.
Content	15	(1, 7-12, 14, 15, 18)	Addressing cultural issues affecting mothers and specifically targeting them.
Concepts	8	(1, 7-12, 16)	Problem was conceptualised and presented to them in a culturally appropriate manner. E.g., Using pictorial formats and physical activities to cater for rural population.
Goals	7	(7-12)	Participants were given goals between the intervention sessions, while keeping cultural appropriation in mind.
Methods	12	(1, 3, 6, 7-13, 16, 17)	culturally appropriate methods used to construct messages based on local beliefs on child development.
Context	9	(1, 7, 9-12, 14, 16) (14, 15, 17)	Using local cultural narrative, idioms, and images, books to explain concepts. E.g. dressing up dolls in Bangladeshi attire.

<u>Supplementary Material S6:</u> <u>Grading Evidence Profile</u>

Table S1. GRADE Evidence Profile: Overall Certainty of Evidence for Culturally Adapted Parenting Interventions.

Domain	Assessment	Explanation
**Study limitations (risk of bias)**	Serious	Several studies had unclear allocation, blinding, and incomplete outcome data.
**Inconsistency**	Serious	Substantial heterogeneity across studies (I^2 often > 90%) and variability in interventions and populations.
**Indirectness**	Not serious	Participants, interventions, and outcomes were generally applicable to the target population.
**Imprecision**	Serious	Many studies had small sample sizes or wide confidence intervals.
**Publication bias**	Undetected/possible	Cannot be fully ruled out due to small number of studies per outcome.

Overall certainty of evidence: *Low*

<u>Supplementary Material S7:</u> <u>Forest Plots</u>

<u>FOREST PLOTS: SENSTIVITY ANALYSIS</u>

Effects of interventions on children's cognitive development by risk of bias ratings.

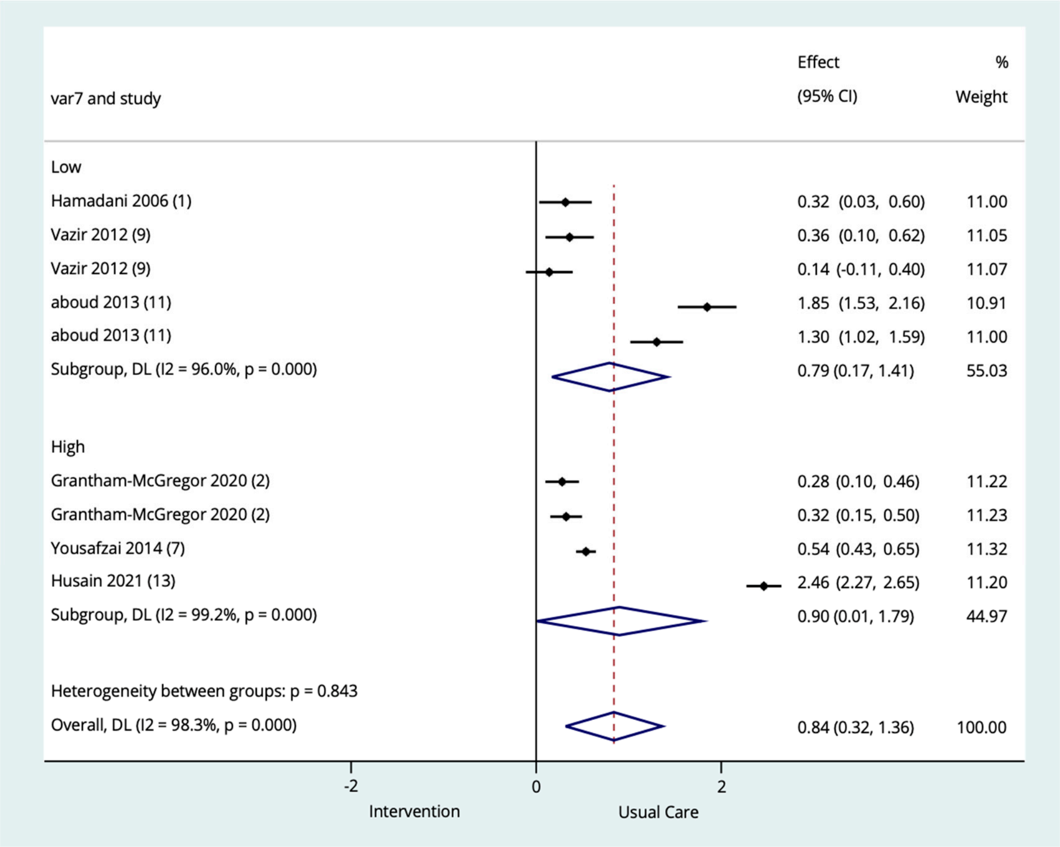

Effects of interventions on child cognitive development by cultural adaptation rating.

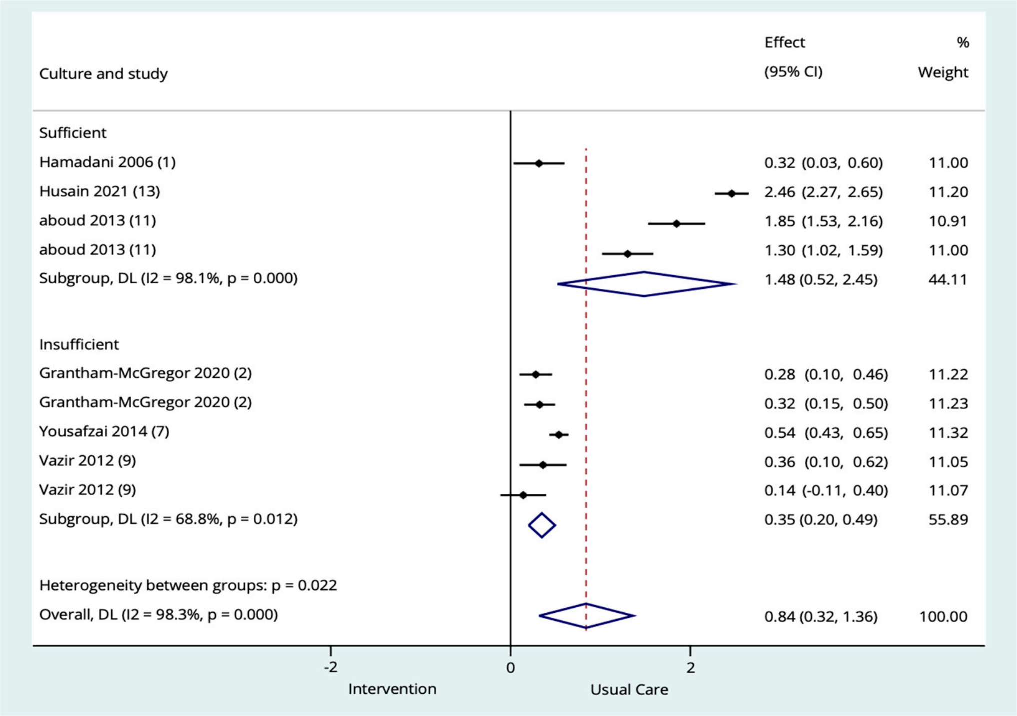

Parenting knowledge:

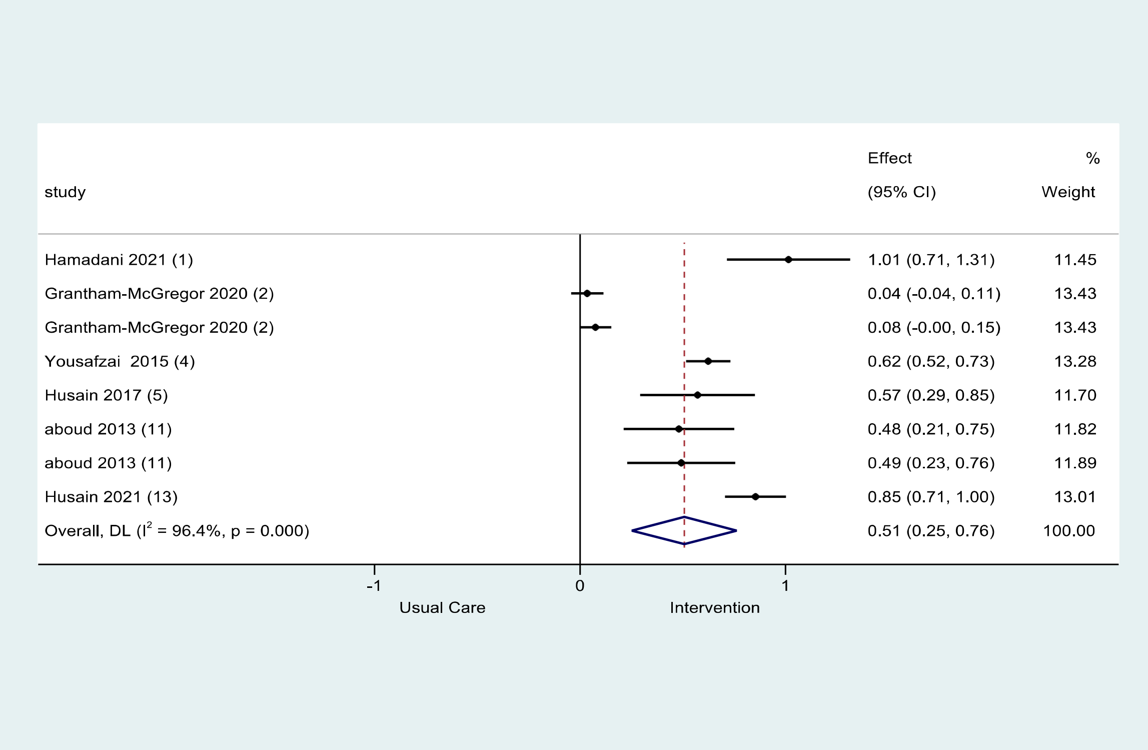

Parental Involvement:

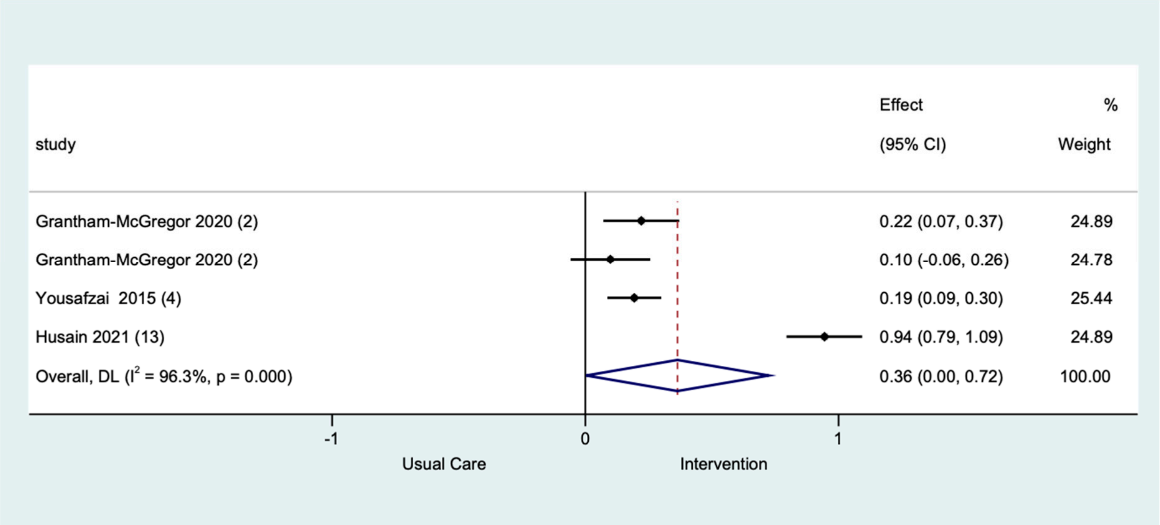

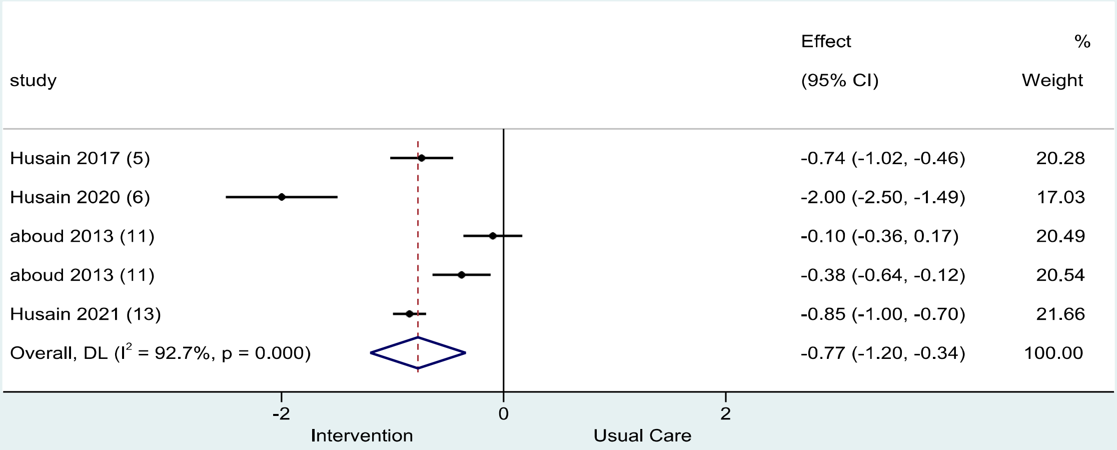
study
Effect (95% CI)
% Weight
Husain 2017 (5) -0.74 (-1.02, -0.46) 20.28
Husain 2020 (6) -2.00 (-2.50, -1.49) 17.03
aboud 2013 (11) -0.10 (-0.36, 0.17) 20.49
aboud 2013 (11) -0.38 (-0.64, -0.12) 20.54
Husain 2021 (13) -0.85 (-1.00, -0.70) 21.66
Overall, DL (I^2 = 92.7%, p = 0.000) -0.77 (-1.20, -0.34) 100.00
-2
0
2
Intervention
Usual Care

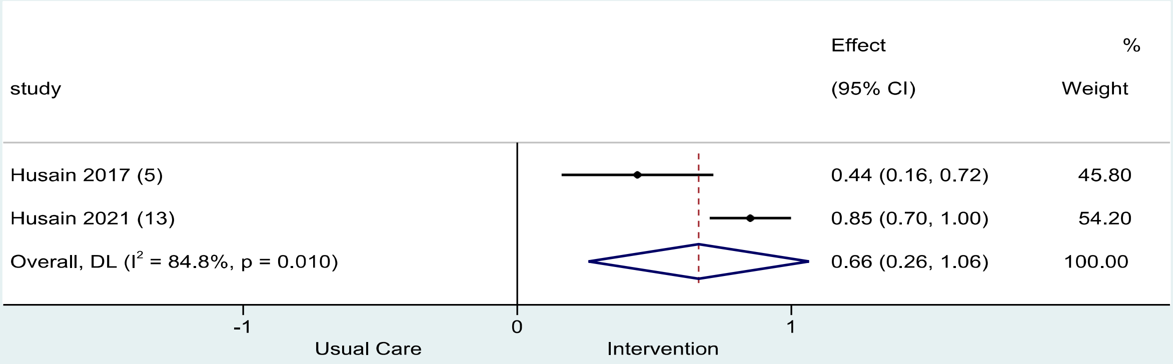
study
Effect (95% CI)
% Weight
Husain 2017 (5) 0.44 (0.16, 0.72) 45.80
Husain 2021 (13) 0.85 (0.70, 1.00) 54.20
Overall, DL (I^2 = 84.8%, p = 0.010) 0.66 (0.26, 1.06) 100.00
-1
0
1
Usual Care
Intervention

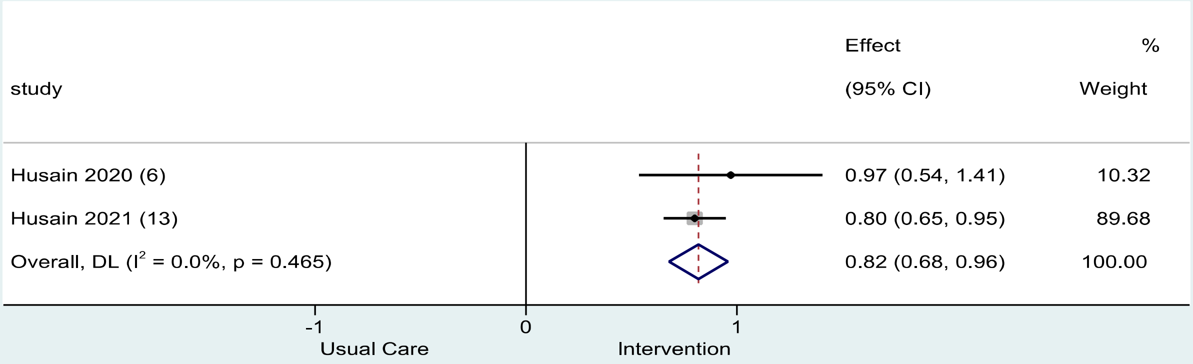
Effect
%
study
(95% CI)
Weight
Husain 2020 (6)
0.97 (0.54, 1.41)
10.32
Husain 2021 (13)
0.80 (0.65, 0.95)
89.68
Overall, DL (I² = 0.0%, p = 0.465)
0.82 (0.68, 0.96)
100.00
-1
0
1
Usual Care
Intervention

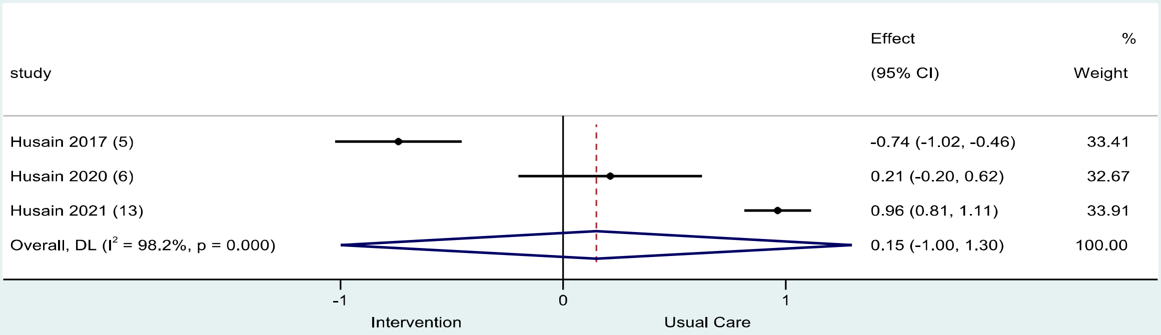
Effect
%
study
(95% CI)
Weight
Husain 2017 (5)
-0.74 (-1.02, -0.46)
33.41
Husain 2020 (6)
0.21 (-0.20, 0.62)
32.67
Husain 2021 (13)
0.96 (0.81, 1.11)
33.91
Overall, DL (I² = 98.2%, p = 0.000)
0.15 (-1.00, 1.30)
100.00
-1
0
1
Intervention
Usual Care

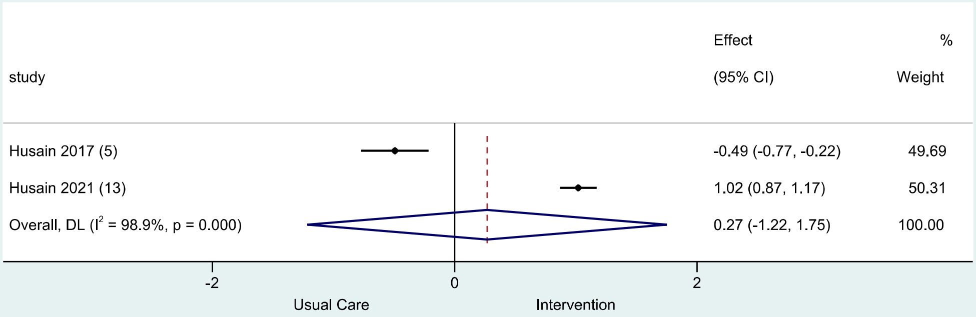
Effect
%
study
(95% CI)
Weight
Husain 2017 (5)
-0.49 (-0.77, -0.22)
49.69
Husain 2021 (13)
1.02 (0.87, 1.17)
50.31
Overall, DL (I² = 98.9%, p = 0.000)
0.27 (-1.22, 1.75)
100.00
-2
0
2
Usual Care
Intervention

CHILD OUTCOMES:

Cognitive outcomes

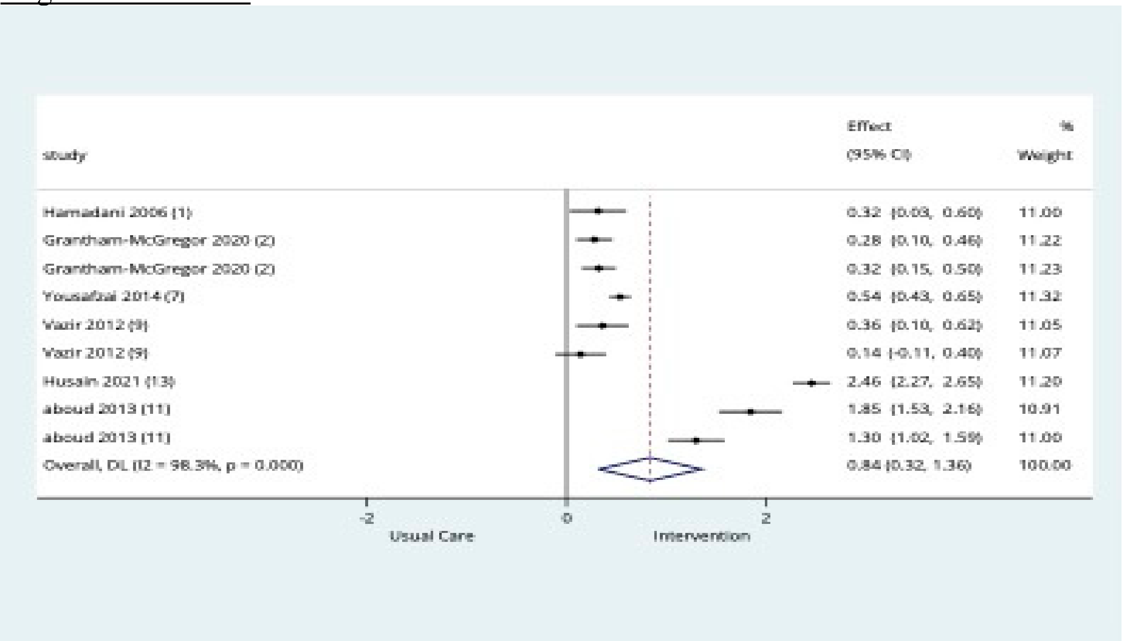

Language development

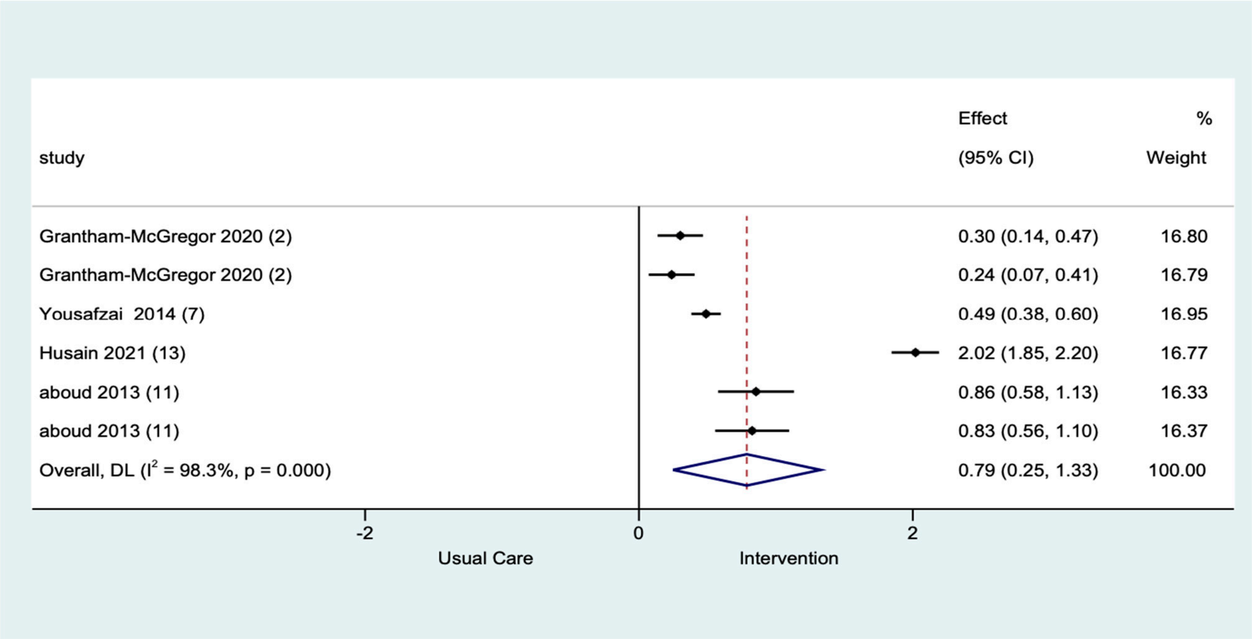

Social development

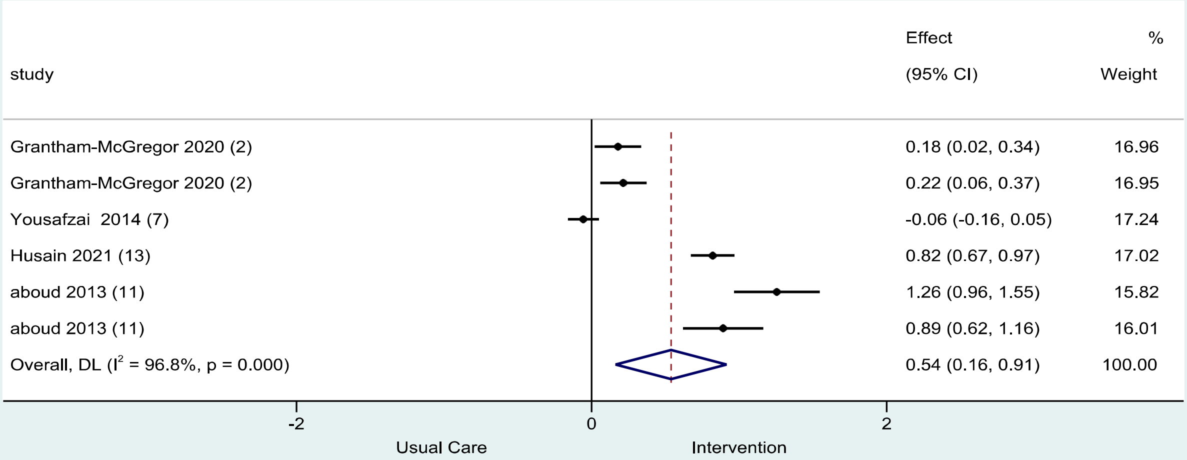

study
Effect
(95% CI)
%
Weight
Grantham-McGregor 2020 (2) 0.18 (0.02, 0.34) 16.96
Grantham-McGregor 2020 (2) 0.22 (0.06, 0.37) 16.95
Yousafzai 2014 (7) -0.06 (-0.16, 0.05) 17.24
Husain 2021 (13) 0.82 (0.67, 0.97) 17.02
aboud 2013 (11) 1.26 (0.96, 1.55) 15.82
aboud 2013 (11) 0.89 (0.62, 1.16) 16.01
Overall, DL (I^2 = 96.8%, p = 0.000) 0.54 (0.16, 0.91) 100.00
-2
0
2
Usual Care
Intervention

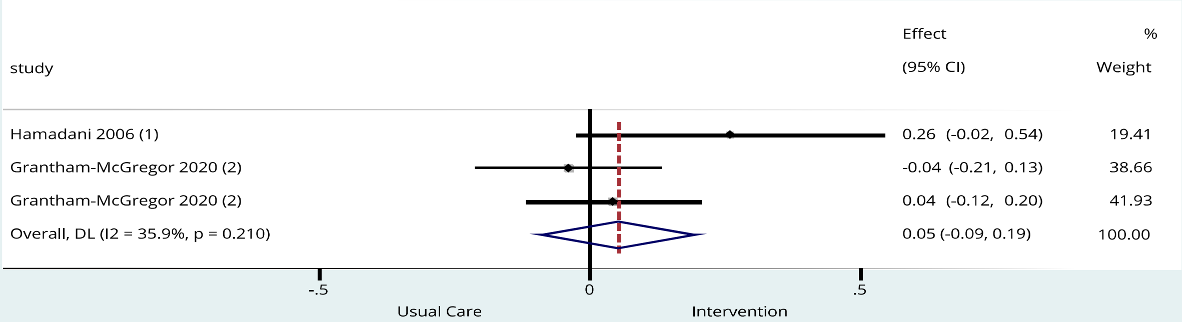

study
Effect
(95% CI)
%
Weight
Hamadani 2006 (1) 0.26 (-0.02, 0.54) 19.41
Grantham-McGregor 2020 (2) -0.04 (-0.21, 0.13) 38.66
Grantham-McGregor 2020 (2) 0.04 (-0.12, 0.20) 41.93
Overall, DL (I2 = 35.9%, p = 0.210) 0.05 (-0.09, 0.19) 100.00
-.5
0
.5
Usual Care
Intervention

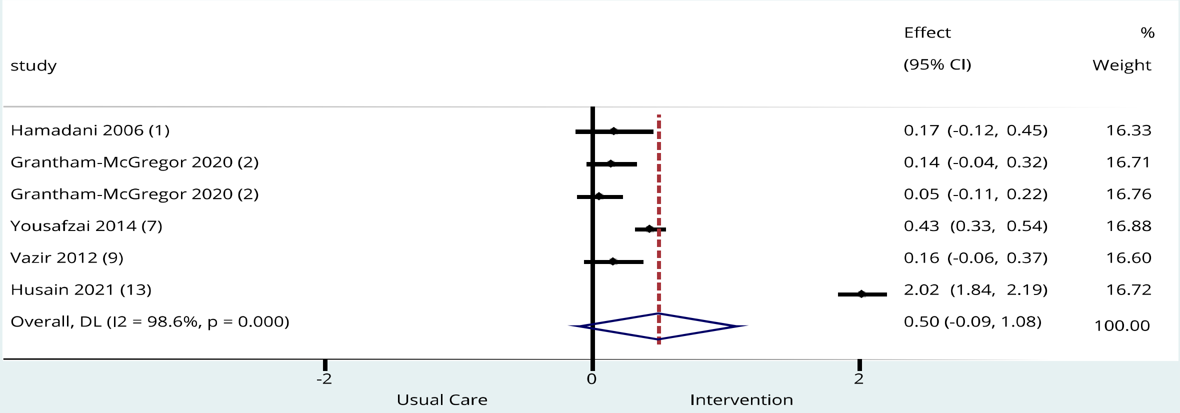

REFERENCES:

1. Hamadani JD, Huda SN, Khatun F, Grantham-McGregor SM. Psychosocial stimulation improves the development of undernourished children in rural Bangladesh. J Nutr. 2006;136(10):2645-52.
2. Grantham-McGregor S, Adya A, Attanasio O, Augsburg B, Behrman J, Caeyers B, et al. Group sessions or home visits for early childhood development in India: a cluster RCT. Pediatrics. 2020;146(6).
3. Ara G, Khanam M, Papri N, Nahar B, Kabir I, Sanin KI, et al. Peer Counseling Promotes Appropriate Infant Feeding Practices and Improves Infant Growth and Development in an Urban Slum in Bangladesh: A Community-Based Cluster Randomized Controlled Trial. Curr Dev Nutr. 2019;3(7):nzz072.
4. Yousafzai AK, Rasheed MA, Rizvi A, Armstrong R, Bhutta ZA. Parenting Skills and Emotional Availability: An RCT. Pediatrics. 2015;135(5):e1247-57.
5. Yousafzai AK, Rasheed MA, Rizvi A, Armstrong R, Bhutta ZA. Effect of integrated responsive stimulation and nutrition interventions in the Lady Health Worker programme in Pakistan on child development, growth, and health outcomes: a cluster-randomised factorial effectiveness trial. Lancet. 2014;384(9950):1282-93.
6. Andrew A, Attanasio O, Augsburg B, Day M, Grantham-McGregor S, Meghir C, et al. Effects of a scalable home-visiting intervention on child development in slums of urban India: evidence from a randomised controlled trial. J Child Psychol Psychiatry. 2020;61(6):644-52.
7. Husain MI, Chaudhry IB, Khoso AB, Wan MW, Kiran T, Shiri T, et al. A group parenting intervention for depressed fathers (LTP+ Dads): A feasibility study from Pakistan. Children. 2021;8(1):26.
8. Rahman A, Iqbal Z, Roberts C, Husain N. Cluster randomized trial of a parent-based intervention to support early development of children in a low-income country. Child: care, health and development. 2009;35(1):56-62.
9. Husain N, Zulqernain F, Carter LA, Chaudhry IB, Fatima B, Kiran T, et al. Treatment of maternal depression in urban slums of Karachi, Pakistan: A randomized controlled trial (RCT) of an integrated maternal psychological and early child development intervention. Asian J Psychiatr. 2017;29:63-70.
10. Husain N, Kiran T, Shah S, Rahman A, Raza Ur R, Saeed Q, et al. Efficacy of learning through play plus intervention to reduce maternal depression in women with malnourished children: A randomized controlled trial from Pakistan(☆). J Affect Disord. 2021;278:78-84.
11. Husain N, Kiran T, Fatima B, Chaudhry IB, Husain M, Shah S, et al. An integrated parenting intervention for maternal depression and child development in a low-resource setting: Cluster randomized controlled trial. Depression and anxiety. 2021;38(9):925-39.
12. Husain N, Chaudhry N, Furber C, Fayyaz H, Kiran T, Lunat F, et al. Group psychological intervention for maternal depression: A nested qualitative study from Karachi, Pakistan. World J Psychiatry. 2017;7(2):98-105.
13. Vazir S, Engle P, Balakrishna N, Griffiths PL, Johnson SL, Creed-Kanashiro H, et al. Cluster-randomized trial on complementary and responsive feeding education to caregivers found improved dietary intake, growth and development among rural Indian toddlers. Matern Child Nutr. 2013;9(1):99-117.
14. Bjørknes R, Larsen M, Gwanzura-Ottemöller F, Kjøbli J. Exploring mental distress among immigrant mothers participating in parent training. Children and Youth Services Review. 2015;51:10-7.
15. Bjørknes R, Manger T. Can parent training alter parent practice and reduce conduct problems in ethnic minority children? A randomized controlled trial. Prevention Science. 2013;14:52-63.

16. Aboud FE, Singla DR, Nahil MI, Borisova I. Effectiveness of a parenting program in Bangladesh to address early childhood health, growth and development. Social Science & Medicine. 2013;97:250-8.
17. Akter F, Rahman M, Pitchik HO, Winch PJ, Fernald LCH, Nurul Huda TM, et al. Adaptation and Integration of Psychosocial Stimulation, Maternal Mental Health and Nutritional Interventions for Pregnant and Lactating Women in Rural Bangladesh. Int J Environ Res Public Health. 2020;17(17).
18. Grantham-McGregor S, Adya A, Attanasio O, Augsburg B, Behrman J, Caeyers B, et al. Group Sessions or Home Visits for Early Childhood Development in India: A Cluster RCT. Pediatrics. 2020;146(6).
19. Husain N, Zulqernain F, Carter L-A, Chaudhry IB, Fatima B, Kiran T, et al. Treatment of maternal depression in urban slums of Karachi, Pakistan: A randomized controlled trial (RCT) of an integrated maternal psychological and early child development intervention. Asian Journal of Psychiatry. 2017;29:63-70.
